# Reasons in favour of universal vaccination campaign against COVID-19 in the pediatric population

**DOI:** 10.1186/s13052-021-01192-4

**Published:** 2022-01-10

**Authors:** Nicola Principi, Susanna Esposito

**Affiliations:** 1 Università degli Studi di Milano, Milan, Italy; 2 Pietro Barilla Children’s Hospital, Department of Medicine and Surgery, University of Parma, Via Gramsci 14, 43126 Parma, Italy

**Keywords:** COVID-19, COVID-19 vaccine, Pediatric infectious disease, SARS-CoV-2

## Abstract

**Background:**

Despite the growing evidence of the extreme efficacy of COVID-19 vaccines in adults and the elderly, the administration of the same prophylactic measures to pediatric subjects is debated by some parents and by a number of researchers. The aim of this manuscript is to explain the reasons for overcoming hesitancy towards COVID-19 vaccination in children and adolescents and to highlight the importance of universal COVID-19 vaccination in the pediatric population.

**Main findings:**

Recent epidemiological data suggest that the risk that a child with COVID-19 is hospitalized or admitted to the pediatric intensive care unit is greater than initially thought. Children may also suffer from long COVID and school closure because of COVID-19 can cause relevant mental health problems in the pediatric population. Placebo-controlled, observer-blinded, clinical trials showed appropriate efficacy, safety and tolerability of authorized mRNA COVID-19 vaccines in children and adolescents 12–17 years old. Vaccination in children younger than 12 years of age will allow further benefits .

**Conclusions:**

COVID-19 vaccine administration seems mandatory in all the children and adolescents because of COVID-19 related complications as well as the efficacy, safety and tolerability of COVID-19 vaccines in this population. Due to the recent approval of COVID-9 vaccines for children 5–10 years old, it is desirable that vaccine opponents can understand how important is the universal immunization against COVID-19 for the pediatric subjects.

## Background

Despite the growing evidence of the extreme efficacy of COVID-19 vaccines in adults and the elderly [1], the administration of the same prophylactic measures to pediatric subjects is strongly debated by many parents and by a number of leading physicians and researchers. Controversies about immunization against COVID-19 in children are almost daily reported in the most largely diffused newspapers and major scientific journals [2–4]. On the other hand, hesitation in recommending immunization against COVID-19 of pediatric subjects has been also demonstrated by health authorities of some countries with advanced health system. After the Food and Drug Administration (FDA) authorization for emergency use of mRNA vaccines in children 12 years of age and older, in the USA both the American Academy of Pediatrics (AAP) and the Centers for Disease Control and Prevention (CDC) have immediately recommended the immunization against COVID-19 of all the children in this age group [5]. On the contrary, in UK immunization against COVID-19 of children 12 to 17 years old was recommended only in case of a severe chronic underlying disease or if they were living with people at risk of very severe COVID-19 [6]. Finally, in Sweden health authorities had a more cautious attitude because the Public Health Agency declared that it was looking at the impact of offering the COVID-19 vaccines to children in other countries before making the decision whether and when to immunize children aged 12 to 15 years [7]. Recently, the FDA [8] and the European Medicines Agency (EMA) [9] have authorized the use of mRNA vaccines in children 5–11 years old. The aim of this manuscript is to explain the reasons for overcoming hesitancy towards COVID-19 vaccination in children and adolescents and to highlight the importance of universal COVID-19 vaccination in the pediatric population.

### Reasons to overcome hesitancy towards COVID-19 vaccination in children and adolescents

The attitude against COVID-19 vaccination of pediatric subjects does not seem to be based on consistent assumptions. Many of the reasons given by those who oppose COVID-19 vaccination in pediatrics do not seem to stand up to a thorough analysis. Opponents argue that mass vaccination of children, while effective in reducing the number of pediatric COVID-19 cases, does not seem to be cost/effective as most severe acute respiratory syndrome coronavirus 2 (SARS-CoV-2) infections in children and adolescents are clinically mild or even asymptomatic [10]. Less than 2% of pediatric cases are hospitalized [11] and, contrarily to adults, death is a rare event [12]. Data collected in 79 countries accounting for 2.7 million COVID-19 deaths have shown that only 0.3% regarded patients younger than 20 years of age [13].

Moreover, those against COVID-19 vaccination in children and adolescents suggested that most of the pediatric cases at risk of severe COVID-19 could be easily identified and this could allow to recommend immunization only to a very reduced number of pediatric patients, avoiding mass vaccination. Some data highlighted that the great majority of severe pediatric COVID-19 cases are diagnosed in children suffering from a severe chronic underlying disease [14] and in those living in countries with greater mortality rates in all age groups [15].

However, a more complete and most up-to-date evaluation of pediatric COVID-19 seems to indicate that the relevance of severe cases in the pediatric population is significantly greater than that indicated by vaccine opponents. Recent epidemiological data seem to suggest that the risk that a child with SARS-CoV-2 infection is hospitalized or admitted to the pediatric intensive care unit (PICU) during the acute phase of the disease is greater than initially thought [16]. In a study involving 20,714 patients 18 years or younger with an emergency department or inpatient encounter for COVID-19, it was shown that 11.7% of these children were hospitalized, 3.6% had a severe disease needing PICU admission and 0.8% were mechanically ventilated [17]. Moreover, in the USA, as of September 23, 2021, over 5.7 million pediatric cases have been diagnosed, with 21,814 hospitalizations and 498 deaths, values that significantly exceeded those usually reported for influenza in an average year and made COVID-19 one of the top 10 causes of death in children in that country [5]. Interestingly, a relevant part of hospitalizations and deaths were due to the multisystem inflammatory syndrome (MIS-C), a condition sometimes very severe that was totally unknown at the beginning of the pandemic [18].

Furthermore, a more careful and prolonged evaluation of pediatric COVID-19 has shown that SARS-CoV-2 infection in the first months/years of life may be potentially associated with an increased risk of long-term alterations. In some cases, neurological symptoms ranging from mild headache to seizure, peripheral neuropathy, stroke, demyelinating disorders, and encephalopathy have been reported. In some cases they are associated with relevant alterations of neuroimaging, electroencephalography, nerve conduction studies and electromyography findings. Despite transient in most children, in some cases neurological damage may become persistent and lead to neurodevelopment delay [19]. Finally, although true incidence, clinical characteristics, severity, duration and long-term effects of the syndrome are not precisely defined, it seems highly likely that children, as adults [20, 21], may suffer from the so-called long COVID. Up to 66% of children with previous SARS-CoV-2 infection, although asymptomatic, were reported to suffer from one of more symptoms for several weeks or months after acute manifestation with reduced quality of life of the patient and his family and a relevant impact on the health system [22].

Importance of COVID-19 vaccine administration in children and adolescents cannot be evaluated only on the base of prevention of COVID-19 clinical manifestations. Prevention of infection, even of asymptomatic cases, is extremely important to reduce circulation of virus among children and transmission of infection from children to unprotected adults [23]. Recent studies have shown that the role of children in transmission of SARS-CoV-2 infection is greater than previously thought [24]. This does not surprise as with the progressive increase of vaccination coverage and protection among adults, children remain a large group of totally susceptible subjects among whom virus freely circulates. Adults living with them can easily become infected. A mathematical model has shown that including adolescents and children in the vaccination program could reduce overall COVID-related mortality by 57%, and reduce cases of long COVID by 75% [25]. In addition, a lower number of SARS-CoV-2 infection among children can limit school closure and related problems, as it has been shown that school closure can cause relevant mental health problems in the pediatric population. Even after few weeks from school closure, depressive symptoms were reported in about 25% of a group of older children and adolescents in China [24]. Sadness and eating problems were evidenced in most of adolescents in Italy [26].

Overall, these factors seem important enough to justify mass vaccination of children of any age,.

### Reasons to support universal vaccination against COVID-19 in children and adolescents

The logic of limiting vaccination only to few children should be rejected. Severe cases of pediatric COVID-19 can occur in otherwise healthy children. Chronic underlying conditions associated with an increased risk of severe COVID-19 are not precisely defined [27]. A more in-depth analysis of the studies evaluating pediatric COVID-19 severity and mortality in the different countries has shown that they have significant methodological limitations because of imperfect and different data collection systems that make comparison totally unreliable. When mortality is referred to the total pediatric population of each country, difference among countries is reduced or almost totally eliminated [28].

Other data evaluated differently by opponents and supporters of COVID-19 vaccination regard efficacy, safety and tolerability of COVID-19 vaccine in children and adolescents. Opponents think that all these aspects have not been adequately evaluated. In their opinion, efficacy has been ascertained on a too small number of subjects. Regarding adverse events, it is highlighted that evaluation has been frequently based on passive surveillance, a method that, particularly for mild to moderate adverse events, largely underestimate real incidence [29]. Moreover, it is highlighted that severe adverse events’ incidence rate can be so high to exceed the potential advantages of vaccination. In a study using data collected by the Vaccine Adverse Event Reporting System (VAERS), it was reported that following mRNA vaccine administration the hospitalization rates of children aged 12–17 years due to cardiac adverse events was several times higher than those expected for COVID-19 in the same age group [30]. In addition, it is pointed out that health authorities have approved authorization of COVID-19 vaccines without third-party access to trial data and documents. Clinical data transparency is essential, as widespread use of interventions without full data transparency raises concerns over the rational use of any therapeutic or prophylactic measure, including COVID-19 vaccines [31].

All the above considerations can be debated. Concerning efficacy and safety, it can be highlighted that health authorities grant authorization for use of COVID-19 vaccines in children and adolescents with extreme caution. Authorizations for emergency use are granted only when results of placebo-controlled, observer-blinded, clinical trials enrolling a few thousand of subjects and showing sufficient efficacy, safety and tolerability of the studied preparations are available and ethical judgment has been obtained. This explains why initially only COVID-19 vaccines for older children and adolescents have been authorized [32, 33] and only later the U.S. FDA and EMA authorized the emergency use of the Pfizer-BioNTech COVID-19 Vaccine for children 5 through 11 years of age [8, 9]. The lack of data transparency is a limitation that must be accepted considering the clinical and socio-economic impact of the pandemic and the need to reduce its consequences as quickly as possible. In addition, as highlighted by the European Pediatric Societies [34], the vaccination in children younger than age 12 years will allow a large number of children to attend school, spend time with friends, travel with their families, and enjoy their communities safely.

Regarding severe adverse events, it cannot be ignored that a number of myocarditis cases, mainly in males and after the second vaccine dose, have been diagnosed in subjects that had received the mRNA vaccines few days before [35] and that data concerning long-term heart problems after COVID-19 immunization are lacking. However, the risk of heart problem development seems significantly lower than that suggested by opponents. The study cited by opponents has an important limitation that makes results highly debatable and inadequate to draw firm conclusions. It is based on VAERS reporting data that simply indicate any safety signal or unexpected pattern of vaccine-related adverse reactions, regardless of believed cause [30]. Information can be inaccurate, erroneous or unverified and do not allow to evaluate presence, prevalence, incidence, and severity of vaccine-related adverse events. A recent assessment by the US Advisory Committee of Immunization Practice of the benefit-risk balance of mRNA vaccines in adolescents and young adults has shown that benefits significantly outweigh the risks. It has been calculated that the administration of the second doses of mRNA COVID-19 vaccines to males aged 12–29 years could prevent 11,000 COVID-19 cases, 560 hospitalizations, 138 intensive care admissions, and 6 deaths, compared with 39–47 expected myocarditis cases after COVID-19 vaccination [36].

All these data show the importance of using COVID-19 vaccines in the pediatric population even when otherwise healthy. Children younger than 12 years of age should be included in the vaccination campaign as soon as health authorities of each country will approve COVID-19 vaccines for their age group.

## Conclusions

COVID-19 vaccine administration seems mandatory in all the children and adolescents because of COVID-19 related complications and efficacy, safety and tolerability of COVID-19 vaccines in this population. It is desirable that vaccine opponents can understand how important is the universal immunization against COVID-19 for the pediatric population. Unfortunately, it will not be easy to obtain a broad parent support to a mass pediatric vaccination. One year after the approval of COVID-19 vaccines, a not marginal part of adults still refuse vaccination. It is clear that vaccination of children will have to be supported by strong persuasion campaign capable of convincing the adults who are still reluctant to accept the vaccine.

## Data Availability

All included.

## Abbreviations

AAP: American Academy of Pediatrics
CDC: Centers for Disease Control and Prevention
COVID-19: New coronavirus disease 2019
FDA: Food and Drug Administration
MIS-C: Multisystem inflammatory syndrome
PICU: Pediatric intensive care unit
SARS-CoV-2: severe acute respiratory syndrome coronavirus
VAERS: Vaccine Adverse Event Reporting System

## References

[1] Self WH, Tenforde MW, Rhoads JP, Gaglani M, Ginde AA, Douin DJ, Olson SM, Talbot HK, Casey JD, Mohr NM, Zepeski A, McNeal T, Ghamande S, Gibbs KW, Files DC, Hager DN, Shehu A, Prekker ME, Erickson HL, Gong MN, Mohamed A, Henning DJ, Steingrub JS, Peltan ID, Brown SM, Martin ET, Monto AS, Khan A, Hough CL, Busse LW, ten Lohuis CC, Duggal A, Wilson JG, Gordon AJ, Qadir N, Chang SY, Mallow C, Rivas C, Babcock HM, Kwon JH, Exline MC, Halasa N, Chappell JD, Lauring AS, Grijalva CG, Rice TW, Jones ID, Stubblefield WB, Baughman A, Womack KN, Lindsell CJ, Hart KW, Zhu Y, Mills L, Lester SN, Stumpf MM, Naioti EA, Kobayashi M, Verani JR, Thornburg NJ, Patel MM, Calhoun N, Murthy K, Herrick J, McKillop A, Hoffman E, Zayed M, Smith M, Seattle N, Ettlinger J, Priest E, Thomas J, Arroliga A, Beeram M, Kindle R, Kozikowski LA, de Souza L, Ouellette S, Thornton-Thompson S, Mehkri O, Ashok K, Gole S, King A, Poynter B, Stanley N, Hendrickson A, Maruggi E, Scharber T, Jorgensen J, Bowers R, King J, Aston V, Armbruster B, Rothman RE, Nair R, Chen JTT, Karow S, Robart E, Maldonado PN, Khan M, So P, Levitt J, Perez C, Visweswaran A, Roque J, Rivera A, Angeles L, Frankel T, Angeles L, Goff J, Huynh D, Howell M, Friedel J, Tozier M, Driver C, Carricato M, Foster A, Nassar P, Stout L, Sibenaller Z, Walter A, Mares J, Olson L, Clinansmith B, Rivas C, Gershengorn H, McSpadden E, Truscon R, Kaniclides A, Thomas L, Bielak R, Valvano WD, Fong R, Fitzsimmons WJ, Blair C, Valesano AL, Gilbert J, Crider CD, Steinbock KA, Paulson TC, Anderson LA, Kampe C, Johnson J, McHenry R, Blair M, Conway D, LaRose M, Landreth L, Hicks M, Parks L, Bongu J, McDonald D, Cass C, Seiler S, Park D, Hink T, Wallace M, Burnham CA, Arter OG, IVY Network, IVY Network (2021). Comparative effectiveness of Moderna, Pfizer-BioNTech, and Janssen (Johnson & Johnson) vaccines in preventing COVID-19 hospitalizations among adults without immunocompromising conditions - United States, march-august 2021. MMWR Morb Mortal Wkly Rep.

[2] The Washington Post. For many families, the countdown has begun to coronavirus vaccines for younger children By Lindsey Bever. Available at: https://www.washingtonpost.com/health/2021/09/27/kids-covid-vaccine/. Accessed 26 Sept 2021.

[3] Ioannidis JPA (2021). COVID-19 vaccination in children and university students. Eur J Clin Investig.

[4] Eberhardt CS, Siegrist CA (2021). Is there a role for childhood vaccination against COVID-19?. Pediatr Allergy Immunol.

[5] Centers for Disease Control and Prevention. CDC director statement on Pfizer’s use of COVID-19 vaccine in adolescents age 12 and older. Available at: https://www-cdc-gov.pros.lib.unimi.it/media/releases/2021/s0512-advisory-committee-signing.html. Accessed 26 Sept 2021.

[6] GOV. UK. JCVI issues advice on COVID- 19 vaccination of children and young people. Available at: https://www.gov.uk/government/news/jcvi-issues-advice-on-covid-19-vaccination-of-children-and-young-people. Accessed 26 Sept 2021.

[7] Sverigesradio. Children aged 12 to 15 to be offered Covid vaccine, government says Available at: https://sverigesradio.se/artikel/children-aged-12-to-15-to-be-offered-covid-vaccine-government-says. Accessed on 26 Sept 2021.

[8] Food and Drug Administration. FDA authorizes Pfizer-BioNTech COVID-19 vaccine for emergency use in children 5 through 11 years of age. Available at: https://www.fda.gov/news-events/press-announcements/fda-authorizes-pfizer-biontech-covid-19-vaccine-emergency-use-children-5-through-11-years-age. Accessed 29 Nov 2021.

[9] European Medicines Agency. Comirnaty COVID-19 vaccine: EMA recommends approval for children aged 5 to 11. Available at: https://www.ema.europa.eu/en/news/comirnaty-covid-19-vaccine-ema-recommends-approval-children-aged-5-11. Accessed 29 Nov 2021.

[10] de Souza TH, Nadal JA, Nogueira RJN, Pereira RM, Brandão MB (2020). Clinical manifestations of children with COVID-19: a systematic review. Pediatr Pulmonol.

[11] American Academy of Pediatrics and the Children’s Hospital Association. Children and COVID-19: state data report. Version: 9/16/21. Available at: https://downloads.aap.org/AAP/PDF/AAP%20and%20CHA%20-%20Children%20and%20COVID-19%20State%20Data%20Report%209.16%20FINAL.pdf. Accessed 27 Sept 2021.

[12] Abu-Raya B, Migliori GB, O'Ryan M, Edwards K, Torres A, Alffenaar JW (2020). Coronavirus disease-19: an interim evidence synthesis of the world association for infectious diseases and immunological disorders (Waidid). Front Med (Lausanne).

[13] MAX Plank Institute COVerAGE-DB: a database of age structured COVID-19 cases and deaths. Available at: https://www.demogr.mpg.de/papers/working/wp-2020-032.pdf. Accessed 27 Sept 2021.

[14] Garazzino S, Lo Vecchio A, Pierantoni L, Calò Carducci FI, Marchetti F, Meini A, Castagnola E, Vergine G, Donà D, Bosis S, Dodi I, Venturini E, Felici E, Giacchero R, Denina M, Pierri L, Nicolini G, Montagnani C, Krzysztofiak A, Bianchini S, Marabotto C, Tovo PA, Pruccoli G, Lanari M, Villani A, Castelli Gattinara G, The Italian SITIP-SIP Pediatric Infection Study Group (2021). Epidemiology, clinical features and prognostic factors of pediatric SARS-CoV-2 infection: results from an Italian multicenter study. Front Pediatr.

[15] COVID-19 INED. The demography of COVID-19 deaths. An unprecedented context of health crisis. Available at: https://dc-covid.site.ined.fr/en/. Accessed 26 Sept 2021.

[16] Esposito S, Marchetti F, Lanari M, Caramelli F, De Fanti A, Vergine G (2021). COVID-19 management in the pediatric age: consensus document of the COVID-19 working group in paediatrics of the Emilia-Romagna Region (RE-CO-Ped), Italy. Int J Environ Res Public Health.

[17] Preston LE, Chevinsky JR, Kompaniyets L, Lavery AM, Kimball A, Boehmer TK, Goodman AB (2021). Characteristics and disease severity of US children and adolescents diagnosed with COVID-19. JAMA Netw Open.

[18] Esposito S, Principi N (2021). Multisystem inflammatory syndrome in children related to SARS-CoV-2. Paediatr Drugs.

[19] Principi N, Esposito S (2021). Are we sure that the neurological impact of COVID 19 in childhood has not been underestimated?. Ital J Pediatr.

[20] Carfì A, Bernabei R, Landi F (2020). Persistent symptoms in patients after acute COVID-19. JAMA..

[21] Huang C, Huang L, Wang Y, Li X, Ren L, Gu X, Kang L, Guo L, Liu M, Zhou X, Luo J, Huang Z, Tu S, Zhao Y, Chen L, Xu D, Li Y, Li C, Peng L, Li Y, Xie W, Cui D, Shang L, Fan G, Xu J, Wang G, Wang Y, Zhong J, Wang C, Wang J, Zhang D, Cao B (2021). 6-month consequences of COVID-19 in patients discharged from hospital: a cohort study. Lancet..

[22] Zimmermann P, Pittet L, Nigel C. How common is long COVID in children and adolescents? [published online ahead of print September 28, 2021]. Pediatr Infect Dis J. Available from: Journals@Ovid Full Text at http://ovidsp.ovid.com/ovidweb.cgi?T=JS&PAGE=reference&D=ovftw&NEWS=N&AN=00006454-900000000-95677. Accessed 04 Oct 2021. 10.1097/INF.0000000000003328 PMC8575095 34870392

[23] Esposito S, Zona S, Vergine G, Fantini M, Marchetti F, Stella M, Valletta E, Biasucci G, Lanari M, Dodi I, Bigi M, Magista AM, Vaienti F, Cella A, Affanni P, Re MC, Sambri V, Principi N, on behalf of the Regione Emilia-Romagna COVID (2020). How to manage children if a second wave of COVID-19 occurs. Int J Tuberc Lung Dis.

[24] Meuris C, Kremer C, Geerinck A, Locquet M, Bruyère O, Defêche J, Meex C, Hayette MP, Duchene L, Dellot P, Azarzar S, Maréchal N, Sauvage AS, Frippiat F, Giot JB, Léonard P, Fombellida K, Moutschen M, Durkin K, Artesi M, Bours V, Faes C, Hens N, Darcis G (2021). Transmission of SARS-CoV-2 after COVID-19 screening and mitigation measures for primary school children attending School in Liège, Belgium. JAMA Netw Open.

[25] Shiri T, Evans M, Talarico CA, Morgan AR, Mussad M, Buck PO (2021). Vaccinating adolescents and children significantly reduces COVID-19 morbidity and mortality across all ages: a population-based modeling study using the UK as an example. Vaccines (Basel).

[26] Esposito S, Giannitto N, Squarcia A, Neglia C, Argentiero A, Minichetti P, Cotugno N, Principi N (2021). Development of psychological problems among adolescents during school closures because of the COVID-19 lockdown phase in Italy: a cross-sectional survey. Front Pediatr.

[27] Esposito S, Caramelli F, Principi N (2021). What are the risk factors for admission to the pediatric intensive unit among pediatric patients with COVID-19?. Ital J Pediatr.

[28] Garcia J, Torres C, Barbieri M, Camarda C, Cambois E, Caporali A, etal (2021). Differences in COVID-19 mortality: implications of imperfect and diverse data collection systems. Population.

[29] Blumenthal KG, Robinson LB, Camargo CA, Shenoy ES, Banerji A, Landman AB, Wickner P (2021). Acute allergic reactions to mRNA COVID-19 vaccines. JAMA..

[30] Høeg TB, Krug A, Stevenson J, Mandrola J. SARS-CoV-2 mRNA vaccination-associated myocarditis in children ages 12–17: a stratified national database analysis. MedRxiv. 2021:2021.08.30.21262866 10.1101/2021.08.30.21262866.

[31] Tanveer S, Rowhani-Farid A, Hong K, Jefferson T, Doshi P (2021). Transparency of COVID-19 vaccine trials: decisions without data. BMJ Evid Based Med.

[32] Frenck RW, Klein NP, Kitchin N, Gurtman A, Absalon J, Lockhart S (2021). Safety, immunogenicity, and efficacy of the BNT162b2 Covid-19 vaccine in adolescents. N Engl J Med.

[33] Ali K, Berman G, Zhou H, Deng W, Faughnan V, Coronado-Voges M (2021). Evaluation of mRNA-1273 SARS-CoV-2 vaccine in adolescents. N Engl J Med.

[34] Pettoello-Mantovani M, Carrasco-Sanz A, Huss G, Mestrovic J, Vural M, Pop TL (2021). Viewpoint of the European Pediatric Societies over Severe Acute Respiratory Syndrome Coronavirus 2 (SARS-CoV-2) vaccination in children younger than age 12 years amid return to school and the surging virus variants. J Pediatr.

[35] Calcaterra G, Mehta JL, de Gregorio C, Butera G, Neroni P, Fanos V, Bassareo PP (2021). COVID 19 vaccine for adolescents. Concern about myocarditis and pericarditis. Pediatr Rep.

[36] Gargano JW, Wallace M, Hadler SC, Langley G, Su JR, Oster M (2021). Use of mRNA COVID-19 vaccine after reports of myocarditis among vaccine recipients: update from the advisory committee on immunization practices - United States, June 2021. MMWR Morb Mortal Wkly Rep.

